# The clinical efficacy and safety of vancomycin loading dose: A systematic review and meta-analysis

**DOI:** 10.1097/MD.0000000000017639

**Published:** 2019-10-25

**Authors:** Hekun Mei, Jin Wang, Haoyue Che, Rui Wang, Yun Cai

**Affiliations:** Center of Medicine Clinical Research, Department of Pharmacy, PLA General Hospital, Beijing, China.

**Keywords:** clinical efficacy, loading dose, trough concentration, vancomycin

## Abstract

**Background:**

The clinical significance of using vancomycin loading dose remains controversial. A systematic review and meta-analysis were performed to assess the clinical efficacy and safety of vancomycin loading dose in the treatment of infections.

**Methods:**

The Pubmed, Embase, Web of Science, and Cochrane Library databases were searched from their inception up to 5 May 2019. Randomized controlled trials (RCTs) and other observational studies were included if they provided clinical outcomes or trough concentrations of vancomycin loading dose (20–30 mg/kg) and conventional-dose (10–20 mg/kg) in the treatment of infections. Achievement of therapeutic concentration (serum trough concentrations of vancomycin reached 15–20 mg/L before the second dose), clinical response (clinical improvement or culture-negative), nephrotoxicity (serum creatinine increase ≥0.5 mg/dL or ≥50% increasing from the baseline), other adverse events (including pruritus, flushing, rash, and/or red man syndrome), and mortality were analyzed. Heterogeneity was identified using the Cochrane *I*^*2*^ statistic, and *P*-value <.10 or *I*^*2*^-values >50% indicated significant heterogeneity. Pooled estimates of the intervention effects were determined by the odds ratios (ORs) and 95% confidence intervals (CIs) in Review Manager program, version 5.3.5.

**Results:**

Two RCTs and 7 cohort studies including 2816 infected patients were selected for the analysis, in which serum trough concentrations of vancomycin following the use of vancomycin loading dose or other outcomes were available. Loading dose group had a significantly higher compliance rate of serum trough concentration of 15 to 20 mg/L (OR = 3.06; 95% CI = 1.15–8.15; *P* = .03) and significantly lower incidence of nephrotoxicity (OR = 0.59, 95% CI = 0.40–0.87; *P* = .008; *I*^*2*^ = 29%) compared with control group. No significant difference was noted between loading dose group and control group in terms of other adverse events and clinical response (OR = 1.98, 95% CI = 0.80–4.93; *P* = .14; *I*^*2*^ = 0%). The use of vancomycin loading doses in patients can indeed increase the achievement of therapeutic concentration.

**Conclusion:**

Vancomycin loading dose increases the achievement of therapeutic concentration without bringing extra risk of nephrotoxicity. However, well-designed large-scale RCTs remain needed to validate the clinical efficacy of vancomycin loading dose and to further evaluate other adverse reactions and mortality.

PROSPERO registration number CRD42018093927

## 1 Introduction

Vancomycin is a type of time-dependent antibiotic used to treat hospital-acquired infections or severe infections caused by susceptible strains of methicillin-resistant *Staphylococci* (MRSA).^[1]^ Its antimicrobial efficacy depends on the time span during which the concentration of vancomycin in serum is higher than the minimal inhibitory concentration (MIC) between 2 administrations. When the concentration of vancomycin reaches 4 to 5 times of the MIC, the antibacterial efficacy is the highest.^[2]^ Studies on animal infection models and clinical pharmacokinetics/pharmacodynamics (PK/PD) show that area-under-the-concentration-time curve over 24 hours/minimum inhibitory concentration (AUC_0–24h_/MIC) is the PK/PD parameter that predicts the clinical and bacteriological efficacy of vancomycin. When AUC_0–24h_/MIC ≥400, bacteria can be cleared up and clinical symptoms can be relieved quickly.^[3]^ However, in clinical practice, AUC_0–24h_/MIC is not routinely available to assess the clinical efficacy of vancomycin. Therefore, trough concentration is often used instead of AUC_0–24h_/MIC to predict the efficacy of vancomycin, because trough concentrations of vancomycin at 15 to 20 mg/L can effectively achieve AUC_0–24h_/MIC ≥400.^[4,5]^ When vancomycin concentration is lower than 10 mg/L, bacteria cannot be effectively removed and vancomycin-resistant bacteria arise, leading to prolonged hospital stay and increased mortality rate.^[6–9]^

Recently, the use of vancomycin has increased due to increased MRSA infection rates. However, many patients were found having sub-therapeutic vancomycin concentrations in serum following initial dosing.^[4]^ At the same time, vancomycin-resistant *Enterococci* and *Staphylococcus aureus* were found in patients. Thus, it is urgent to reconsider the dose of vancomycin in clinical application and use it more effectively. To rapidly reach an effective therapeutic concentration of vancomycin and optimize AUC_0–24h_/MIC, a vancomycin loading dose of 25 to 30 mg/kg (based on actual body weight) in adults and 20 to 25 mg/kg in children is recommended for critically ill patients.^[10]^ However, the safety and effectiveness of vancomycin loading doses in clinical applications remain to be fully evaluated. The purpose of this systematic review and meta-analysis was to systematically assess the currently available data in literatures regarding clinical applications of vancomycin loading dose to validate the clinical efficacy of vancomycin loading dose in the treatment of infections and to provide a reference for clinical medication.

## 2 Methods

### 2.1 Literature search

The Pubmed, Embase, Web of Science, and Cochrane Library databases were searched from their inception up to 5 May 2019. The terms used for the search were “vancomycin” and “loading dose.” In addition, the references of the initially identified articles, including relevant review papers, were also manually screened for related articles. No language restrictions were applied for literature search.

### 2.2 Study selection

The protocol of this study can be found at PROSPERO with the registration number of CRD42018093927.^[11]^ The preferred reporting items for systematic reviews and meta-analysis statement were strictly followed. Randomized controlled trials (RCTs) and cohort studies that provided serum trough concentrations of vancomycin following the use of vancomycin loading dose or other outcomes in the treatment of infections were considered eligible. Studies that focused on pre-clinical research, laboratory research, and epidemiology were excluded. Studies in which the control group or loading dose group included 10 patients or fewer, studies on oral vancomycin use or nonhuman data, and studies that lacked the control group for standard use of vancomycin were also excluded. Two investigators (HKM and JW) independently carried out the literature search and study selection. The disagreements between the 2 investigators were resolved by consulting a third investigator (YC). Final consensus was obtained among all investigators.

### 2.3 Data extraction and evaluation

The 2 investigators (HKM and JW) independently extracted the relevant data and assessed the risk of bias. The following data were extracted from each study:

(1) author(s) and year of publication;
(2) country;
(3) type of study;
(4) baseline characteristics of the population;
(5) type of infection;
(6) number of patients enrolled;
(7) dosing regimen (including loading dose and comparator);
(8) efficacy;
(9) reported adverse effects.

The modified Newcastle–Ottawa scale (NOS) was used to evaluate the quality of each included studies.^[12]^ Studies with a NOS score <3 were classified as poor quality studies and excluded from this meta-analysis. Studies were ranked on the basis of their quality of evidence according to the US preventive Services Task 1996 rating system.^[13]^ Level I studies were RCTs. Level II-1 studies were controlled studies, with patients acting as their own controls or with a parallel control group. Level II-2 studies were cohort or case-control designs. Level II-3 studies were multiple time series or exceptional descriptive articles. Level III studies were expert opinion, descriptive studies, and case reports.

### 2.4 Statistical analysis

Achievement of therapeutic concentration was the primary outcome we assessed. The secondary outcomes were: clinical response, nephrotoxicity, other adverse events, and mortality.

All statistical analyses were carried out using the Review Manager program, version 5.3.5 (Cochrane Collaboration, Oxford, United Kingdom). The heterogeneity of the results from included studies was assessed using Chi-square test of heterogeneity and the *I*^*2*^ measure of inconsistency. A Mantel–Haenszel random-effect model was used to assess the odds ratios (ORs) and 95% confidence intervals (CIs) for all primary and secondary outcomes throughout the meta-analysis. Heterogeneity was investigated through subgroup analysis, as defined above. *P*-values <.05 were considered statically significant. Since the present study is based on the published data, ethical approval and informed consent are not applicable.

## 3 Results

### 3.1 Literature search

**Figure 1:**
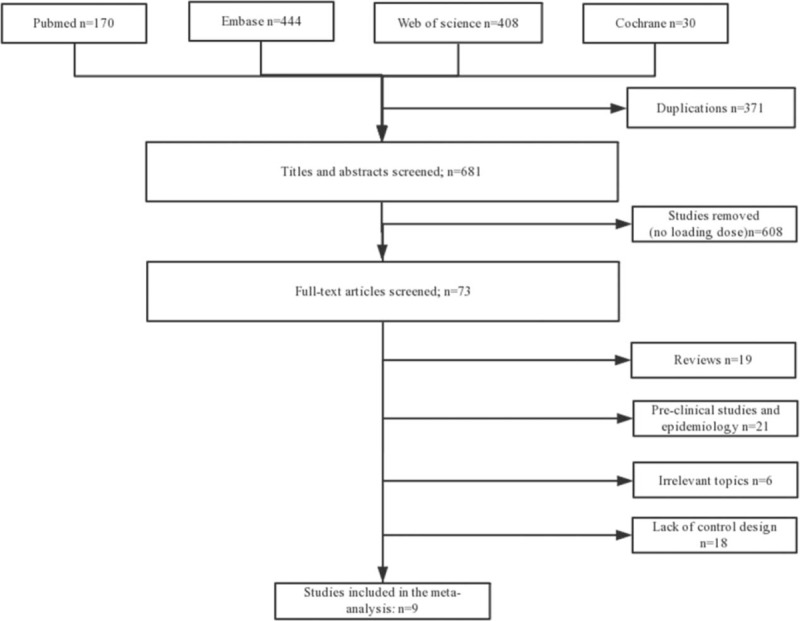
Flowchart of the article selection process.

After literature search, we identified 1052 articles from 4 databases and another 4 articles from the references. In total, 73 relevant full-text articles were screened. In these articles, 19 reviews were excluded from the analysis because they were not original studies. In the remaining 54 articles, 45 articles were excluded, including 21 articles on pre-clinical studies and epidemiology, 18 studies lacking a control group, and 6 studies on irrelevant topics. Finally, 2 RCTs (158 patients) and 7 other studies (2658 patients) were included in the systematic review and meta-analysis.^[14–22]^ The literature search and study selection process are shown in Figure 1.

### 3.2 Study characteristics

**Figure 2:**
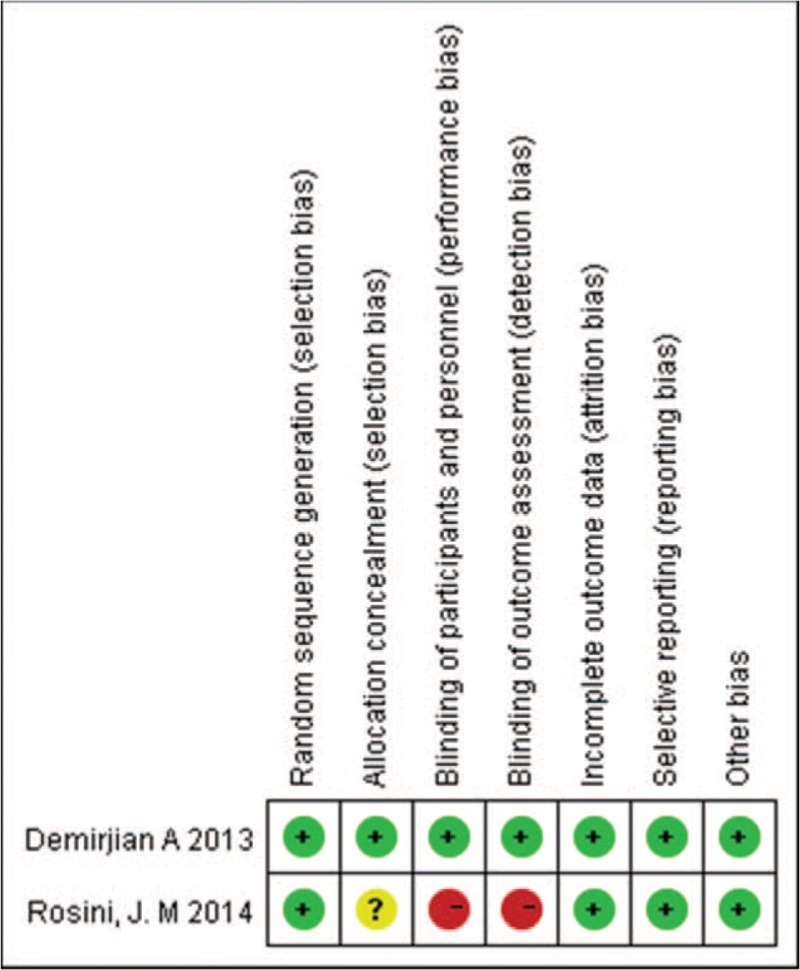
Risk of bias item for each included RCTs. RCTs = randomized controlled trials.

The main characteristics of the included studies are shown in Table 1 . The quality of the 2 RCTs was evaluated by the Cochrane risk of bias tool, and the results showed that the quality of these 2 RCTs was pretty high (Fig. 2). One of the RCTs that was carried out in children aged 2 to 18 years was double-blind study, and the other RCT studied the application of loading dose in emergency department patients. Among the rest 7 studies, 2 of the observational studies were prospective cohort studies, 1 study conducted both retrospective and prospective evaluation and compared the results with each other. The subjects of 2 included studies were critically ill patients. Other studies included intensive care unit (ICU) patients, sepsis patients, patients with severe renal impairment, emergency department patients, hospitalized hemodialysis patients, and MRSA infected patients. The quality of the included studies was assessed by NOS, and the results showed that most studies had a score of 7 to 9 (Table 2).

**Table 1:**
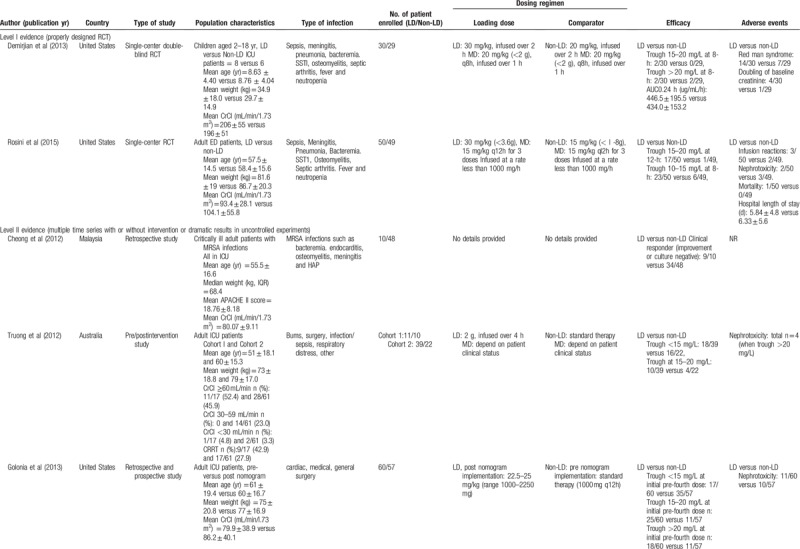
Main characteristics of the studies included in the meta-analysis.

**Table 1 (Continued):**
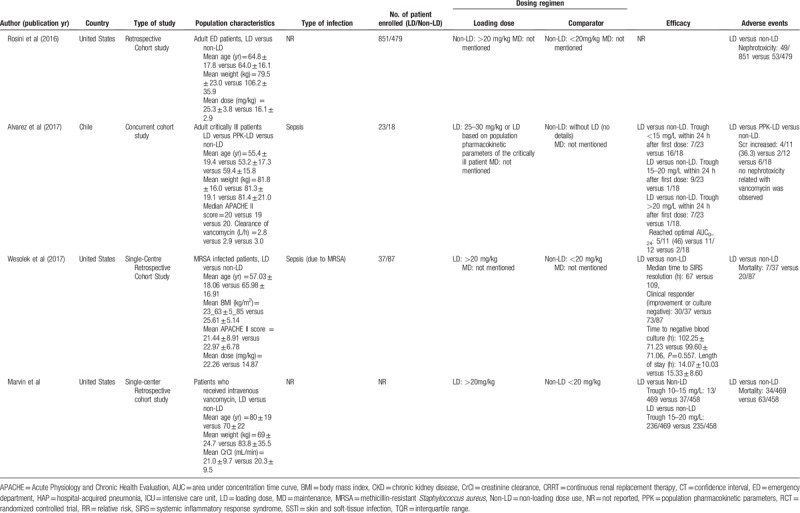
Main characteristics of the studies included in the meta-analysis.

**Table 2:**
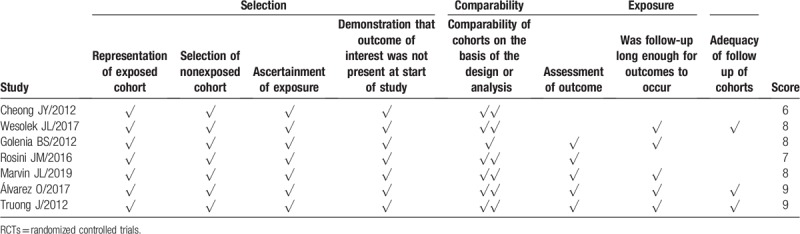
Newcastle–Ottawa scale for assessing the quality of included non-RCTs.

### 3.3 Achievement of therapeutic concentration

**Figure 3:**
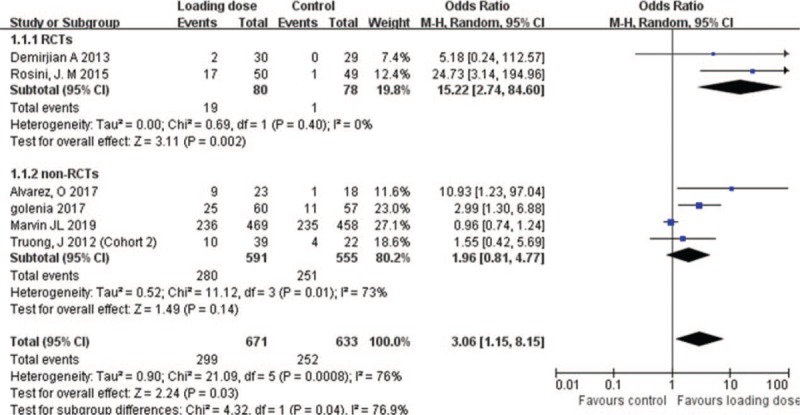
Achievement of therapeutic concentration with vancomycin loading dose versus control.

As shown in Figure 3, the overall achievement of therapeutic concentration was significantly higher in loading dose group than in control group (6 studies; 1304 patients; OR = 3.06 [95% CI = 1.15–8.15]; *P* = .03; *I*^*2*^ = 76%). In RCTs subgroup, the number of cases that achieved 15 to 20 mg/L vancomycin serum trough concentration was significantly higher in loading dose group than in the control (OR = 15.22; 95% CI = [2.74–84.60]; *P* = .002; *I*^*2*^ = 0%). In non-RCTs subgroup, the number of cases that achieved the target trough concentration of vancomycin was also higher in loading dose group than in control group, but the difference was not statistically significant (OR = 1.96; 95% CI = [0.81–4.77]; *P* = .14; *I*^*2*^ = 73%).

### 3.4 Nephrotoxicity

**Figure 4:**
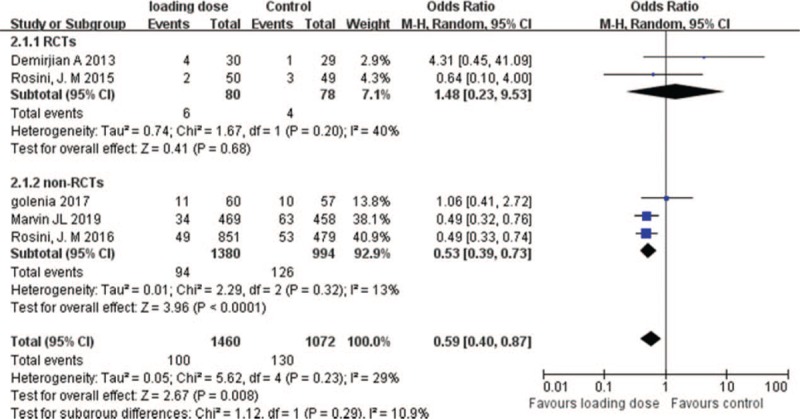
Nephrotoxicity with vancomycin loading dose versus control.

Two RCTs and 3 cohort studies (2532 patients) compared vancomycin exposure-associated nephrotoxicity between vancomycin loading dose group and the control group. The number of patients who suffered from nephrotoxicity was significantly lower in loading dose group than in control group (OR = 0.59; 95% CI = [0.40–0.87]; *P* = .008; *I*^*2*^ = 29%) and analysis of non-RCTs subgroups also showed significantly lower incidence in loading dose group (OR = 0.53; 95% CI = [0.39–0.73]; *P* < .0001, *I*^*2*^ = 13%). However, no significant differences were identified between vancomycin loading dose group and the control group in RCTs subgroups (OR = 1.48; 95% CI = [0.23–9.53]; *P* = .68; *I*^*2*^ = 40%) (Fig. 4).

### 3.5 Other adverse events

**Figure 5:**
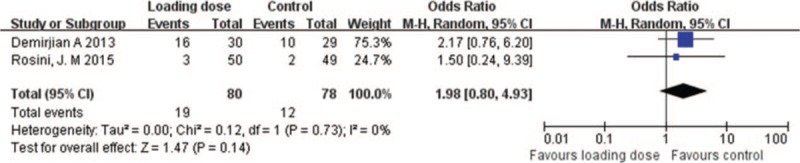
Other adverse events with vancomycin loading dose versus control.

In addition to nephrotoxicity, the common adverse events of vancomycin exposure also included pruritus, flushing, and/or rash. In the included studies, only 2 RCTs (158 patients) compared other adverse events between vancomycin loading dose group and control group, and no significant differences were observed between the 2 groups (OR = 1.98; 95% CI = [0.80–4.93]; *P* = .14; *I*^*2*^ = 0%) (Fig. 5). In most cases, vancomycin was infused over 1 hour or at a rate less than 1000 mg/h to prevent red man syndrome and other adverse events (Table 2).

### 3.6 Mortality

**Figure 6:**
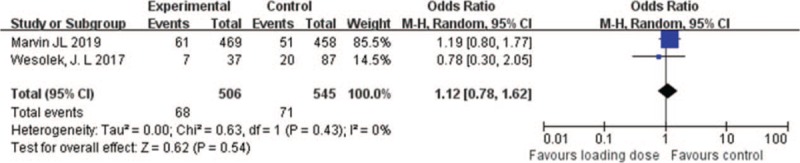
Mortality with vancomycin loading dose versus control.

Two studies reported the mortality following vancomycin loading dose administration (1051 patients), and the results showed that mortality was not significantly different between loading dose group and control group (OR = 1.12; 95% CI = [0.78–1.62]; *P* = .54; *I*^*2*^ = 0%) (Fig. 6).

### 3.7 Clinical response

**Figure 7:**
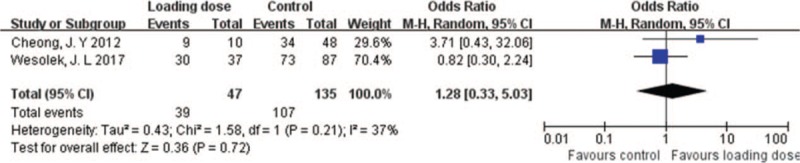
Clinical response with vancomycin loading dose versus control.

The clinical response referred to a response of the patient to vancomycin administration. Patients who responded clinically with negative bacterial culture at the end of therapy and those who responded clinically but without culture result at the end of therapy were all considered to have clinical response. Only 2 cohort studies reported negative blood culture or clinical response. The clinical response was numerically higher in loading dose group than in control group. However, no significant difference was found between the 2 groups (OR = 1.28; 95% CI = [0.33–5.03]; *P* = .72; *I*^*2*^ = 37%) (Fig. 7).

## 4 Discussion and conclusions

According to the United States Food and Drug Administration, the section of dosage and administration, the normal daily intravenous dose of vancomycin is 2 g divided either as 500 mg every 6 hours or 1 g every 12 hours for patients with normal renal function. The label of Vancomycin Injection approved by FDA does not mention the usage of vancomycin loading dose.^[23]^ However, in several clinical practice guidelines published in Japan and the United States, loading dose is recommended for seriously ill patients with suspected MRSA infection (eg, those with sepsis, meningitis, pneumonia, or infective endocarditis).^[4,6]^ Several studies^[16,21]^ reported better clinical outcomes in patients treated with vancomycin loading dose. However, due to the lack of large size samples and well-controlled design, these studies were not enough to confirm the efficacy and safety of vancomycin loading dose. Adequate studies and statistical analysis remain needed to support the clinical efficacy of vancomycin loading dose. The results of our systematic review and meta-analysis indicate that vancomycin loading dose therapy may be a better treatment for patients who have serious infections caused by susceptible strains of MRSA, compared with nonloading dose therapy. To our knowledge, this is the first systematic review to assess the clinical efficacy and safety of vancomycin loading dose.

We found that loading dose group can achieve optimal trough concentration significantly better than nonloading dose group in pooled studies. Soto et al^[24]^ found that standard vancomycin dose of 500 mg every 6 hours is sub-therapeutic in critically ill patients. Mohammedi's^[25]^ results suggest that 15 mg/kg vancomycin loading dose should be considered in critically ill patients with suspected Gram-positive infections. In most cases, when the loading dose is used, the optimal trough concentrations of 15 to 20 mg/L can be achieved in adults within 24 hours before the second dose, no matter the dosing interval is 6, 8, or 12 hours. As for children, Demirjian's study^[15]^ shows that the compliance rate of optimal trough concentration is higher in loading dose group than in control group, but the difference was not statistically significant. Regarding hospitalized hemodialysis patients, Nekidy et al^[26]^ conducted a prospective observational cohort study in adult patients with chronic kidney disease who were hospitalized for hemodialysis. They found that among 24 patients who achieved the recommended pre-hemodialysis serum vancomycin concentration (15–20 mg/L), 14 patients received a loading dose of 15 to 20 mg/kg. The finding of this study favors the use of vancomycin loading dose of 15 to 20 mg/kg. Previously, Wang^[27]^ reported that loading dose can accelerate the build-up of vancomycin serum trough concentration to above 8 mg/L within the first 24 to 48 hours to ensure the best therapeutic outcome.

Nephrotoxicity is one of the main adverse reactions of vancomycin. Considering that the loading dose may bring a higher risk of nephrotoxicity, it is uncertain whether the vancomycin loading dose is safe for clinical application. Our meta-analysis showed that nephrotoxicity was even lower in the loading dose group than in control group, indicating that the loading dose does not associate with increased nephrotoxicity. We found that loading dosage is only associated with the reduction of nephrotoxicity in non-RCTs subgroup, while no significant difference was found in RCTs group. There may be 2 reasons for this result. First, in the non-RCTs studies, clinicians may be able to pay more attention to the patients receiving loading dose and take renal protective measures. However, the relevant data are not available from the original studies. Second, most of the patients in non-RCTs studies were ICU patients and sepsis patients with poor basic conditions and may have a rapid progress of disease. In this case, the decline in renal function is more likely to be related to the progression of the disease. Compared with conventional-dose, loading dose of vancomycin can ensure the rapid achievement of target drug concentration and produce better antibacterial results. Effective control of infection might delay the progression of renal damage.

Higher dose of vancomycin is also considered to cause other adverse effects. Our meta-analysis indicated that the occurrence rate of adverse events in loading dose group was not significantly higher than that of the control group. FDA advised that vancomycin should be administered over a period of at least 60 minutes to avoid rapid-infusion-related reactions. For patients’ safety, prolonged infusion time is preferred when using vancomycin loading dose. For example, Rosini et al^[15]^ recommended that vancomycin should be infused at a rate less than 1000 mg/h. Demirjian et al^[14]^ suggested that vancomycin should be infused over 1 hour to prevent infusion-related reactions. With prolonged infusion time, the incidence of infusion-related reactions seems to be reduced in loading dose group. The bactericidal effect of vancomycin is time-dependent and has long post-antibiotic effect. Thus, maintaining an effective trough concentration can improve the bactericidal efficacy. Vuagnat et al^[28]^ conducted a cohort study to compare the efficacy and safety of intermittent vancomycin infusion (IVI) and continuous vancomycin infusion (CVI) in high-dose therapy. With respect to pharmacokinetics, the plateau concentration of vancomycin in CVI group was significantly higher than the mean trough vancomycin concentration in IVI group. Therefore, prolonged infusion with loading dose may increase trough concentration of vancomycin compared with normal infusion time.

In addition, there was no significant difference in mortality and clinical response between the traditional dose group and the load dose group, which also confirmed that the loading dose of vancomycin is safe, and could be used as an alternative treatment for bacterial infection.

The present systematic review had some limitations and should be viewed prudently. First, only 2 RCTs were included with limited number of cases, and more RCTs are needed to validate the results. Second, some outcomes of included studies were not provided. For example, only 2 studies reported the clinical response and mortality, which may reduce the power of the statistical analysis. In addition, heterogeneity existed in some of the analyses, even when subgroup analysis was conducted.

In conclusion, the use of vancomycin loading doses can significantly increase the achievement of therapeutic concentration. In terms of safety, loading dose reduces the incidence of nephrotoxicity and does not increase the incidence of other adverse events. Based on the available data, loading dose of vancomycin is proved to be a safe and effective therapeutic option for critically ill patients. High-quality large-scale RCTs remain needed to further validate the safety and efficacy of vancomycin loading dose.

## Author contributions

**Conceptualization:** Rui Wang, Yun Cai.

**Data curation:** Hekun Mei, Jin Wang, Haoyue Che, Rui Wang, Yun Cai.

**Formal analysis:** Hekun Mei, Jin Wang, Haoyue Che, Rui Wang, Yun Cai.

**Supervision:** Yun Cai.

**Writing – original draft:** Hekun Mei, Jin Wang, Haoyue Che, Yun Cai.

**Writing – review and editing:** Hekun Mei, Jin Wang, Haoyue Che, Rui Wang, Yun Cai.

Haoyue Che orcid: 0000-0001-8784-3972.
